# Death registration in Nigeria: a systematic literature review of its performance and challenges

**DOI:** 10.1080/16549716.2020.1811476

**Published:** 2020-09-07

**Authors:** Olusesan Ayodeji Makinde, Clifford Obby Odimegwu, Mojisola O. Udoh, Sunday A. Adedini, Joshua O. Akinyemi, Akinyemi Atobatele, Opeyemi Fadeyibi, Fatima Abdulaziz Sule, Stella Babalola, Nosakhare Orobaton

**Affiliations:** a Viable Helpers Development Organization, Abuja, Nigeria; b Viable Knowledge Masters, Abuja, Nigeria; c Demography and Population Studies Program, Schools of Public Health and Social Sciences, University of the Witwatersrand, Johannesburg, South Africa; d Department of Pathology, University of Benin/University of Benin Teaching Hospital, Benin-City, Nigeria; e Vaccine and Infectious Disease Analytics Research Unit, University of the Witwatersrand, Johannesburg, South Africa; f Department of Epidemiology and Medical Statistics, College of Medicine, University of Ibadan, Ibadan, Nigeria; g Monitoring and Evaluations Unit, United States Agency for International Development, Abuja, Nigeria; h Health Department, World Bank, Abuja, Nigeria; i Bloomberg School of Public Health, Johns Hopkins University, Baltimore, MD, USA; j MNCH Program Strategy Team, Bill and Melinda Gates Foundation, Seattle, WA, USA

**Keywords:** Civil registration, death certificates, death registration, demography, governance, health planning, outcome evaluation, sustainable development goals, vital statistics

## Abstract

**Background:**

Death registration provides an opportunity for the legal documentation of death of persons. Documentation of deaths has several implications including its use in the recovery of inheritance and insurance benefits. It is also an important input for construction of life tables which are crucial for national planning. However, the registration of deaths is poor in several countries including Nigeria.

**Objective:**

This paper describes the performance of death registration in Nigeria and factors that may affect its performance.

**Methods:**

We conducted a systematic literature review of death registration completeness in Nigeria to identify, characterize issues as well as challenges associated with realizing completeness in death registration.

**Results:**

Only 13.5% of deaths in Nigeria were registered in 2007 which regressed to 10% in 2017. There was no data reported for Nigeria in the World Health Organization database between 2008 and 2017. The country scored less than 0.1 (out of a maximum of 1) on the Vital Statistics Performance Index. There are multiple institutions with parallel constitutional and legal responsibilities for death registration in Nigeria including the National Population Commission, National Identity Management Commission and Local Government Authorities, which may be contributing to its overall poor performance.

**Conclusions:**

We offer proposals to substantially improve death registration completeness in Nigeria including the streamlining and merger of the National Population Commission and the National Identity Management Commission into one commission, the revision of the legal mandate of the new agency to mainly coordination and establishment of standards. We recommend that Local Government authorities maintain the local registries given their proximity to households. This arrangement will be enhanced by increased utilization of information and communications technology in Civil Registration and Vital Statistics processes that ensure records are properly archived.

## Background

According to the United Nations Statistics Division, a Vital Statistics System is ‘the total process of a) collecting information by civil registration or enumeration on the frequency of occurrence of specified and defined vital events as well as relevant characteristics of the events themselves and of the person or persons concerned and b) compiling, processing, analyzing, evaluating, presenting and disseminating these data in statistical form’ [1]. Civil registration is the main source of data consumed in the Vital Statistics System. Civil Registration ‘involves the continuous gathering of information on all relevant vital events occurring within the boundaries of a country’ [1].

Death is one of the vital events recorded in a Civil Registration and Vital Statistics (CRVS) System and its documentation is the basis for the legal approval for the burial or other disposal of deceased individuals [1,2]. Other benefits of death registration include: veritable source of data for epidemiological investigations, recovery of inheritance and insurance claims, to substantiate an assertion of death and to avoid problems with law enforcement agents in case of need for conveyance of a corpse [3]. In accord with laws of many countries including Nigeria, unnatural causes of deaths (including, homicides, suicides, accidents, or death by misadventure) require investigation by the state [4,5]. Inadequate documentation and certification of death by natural causes (resulting from internal malfunctioning of the body) may delay timely public health interventions such as in situations where death arises from an evolving disease outbreak. Furthermore, underreporting of death from unnatural causes may result in delay or denial of justice.

Despite its prominent importance as articulated in policy documents in many countries, CRVS has not received the attention it deserves, especially in low- and middle-income countries (LMIC) [1]. Lack of progress in improving CRVS has been labeled as the single most critical failure of development leading to a scandal of invisibility, and resulting in people passing through the world without ever being documented [6]. Whilst all vital events are underreported, the registration of deaths is the most under-developed when contrasted with the registration of births and marriages. With the exception of the island countries of Mauritius and Seychelles, no other African country has achieved a complete and functioning CRVS system [7]. The CRVS is seen as the gold standard for addressing the statistical variations introduced by different surveys and methods. However, most African countries have over-relied on periodic household surveys for births and deaths statistics [8]. Death registration is observed to have improved since the 1980s when one-third of deaths were registered to about two-thirds registered between 2005 and 2009 [9]. Researchers are adopting innovative strategies to improve cause of death determination. For instance, researchers and public health specialists are utilizing minimally invasive tissue sampling (MITS), a postmortem diagnostic procedure, together with verbal autopsy to improve mortality reporting and determination of cause of death in low- and middle-income countries where child mortality remains high [10–12]. However, underreporting is still prevalent especially in low-income countries where about 1% of deaths are registered [13,14]. As of 2015, of 46 African countries evaluated, 42 had not yet reported credible death registration data to the United Nations [3]. Amongst the cluster of institutions of the United Nations co-responsible for assisting countries to strengthen death registration systems, roles are neither clear nor well delineated, and may be limiting their effectiveness [14,15].

Measuring the performance of country CRVS systems is a challenge [16]. A composite indicator – the Vital Statistics Performance Index (VSPI) – was developed as a standardized metric to assess the performance of the CRVS across the world [17]. The VSPI assesses the performance of the CRVS by proxy using mortality data. The index consists of six dimensions: completeness of death reporting, quality of cause-of-death reporting, level of cause-specific detail, internal consistency, quality of age and sex reporting, and data availability or timeliness [17]. The global VSPI analysis showed that high-income countries in Europe, America, and Australia have the best performing CRVS systems. However, countries such as Mexico, Moldova, and Serbia without similar resources equally achieved high CRVS completeness rates, suggesting that performance is not entirely dependent on the wealth of a country [9].

To date, Nigeria has relied on estimations for the determination of maternal mortality ratios which have varied across different studies thereby resulting in confusion [18]. Given the underperformance of Nigeria’s CRVS, the objective of this paper is to utilize insights from a systematic literature review of death registration in Nigeria to identify and describe factors associated with realizing completeness in death registration. We propose recommendations and solutions to substantially improve death registration completeness in Nigeria at national and subnational levels.

## Role of death registration in national planning in Nigeria

Death registration and medical certification of cause of death according to the International Statistical Classification of Diseases and Other Related Health Problems provide data on the causes of mortality and by extension the burden of diseases in an environment [8,16]. Used in combination with routine health information systems, it provides excellent evidence-based planning resources for the health system [19]. Years-of-lives lost to a disease and quality-adjusted life years are important healthcare quality indicators that can only be properly determined where the CRVS including death registration is optimal. Evidence has shown that countries with optimally functioning civil registration systems generally have health outcomes that outperform countries with suboptimal CRVS [20]. Scandinavian countries demonstrate how an efficient CRVS system can gradually reduce and eliminate the need for repeated censuses thereby saving costs and providing continuous monitoring data for the health system and for other planning purposes [21].

### Socio-economic, demographic and epidemiological importance of death registration

With the rising prevalence of non-communicable diseases as part of epidemiological transitions, several LMIC countries are beset with the need to document the causes of death and the age at death for research purposes, is also another important reason for death registration. Improved death registration in such a context improve countries’ capacity to measure and track ‘the complex change in patterns of health and disease and on the interactions between these patterns and their demographic, economic and sociologic determinants and consequences’ [22,p.732]. The transition of disease patterns requires investigation to ascertain risk factors for diseases and appropriate mitigation measures. Whereas countries and geographic areas will experience transition differently on account of differences in context, it remains crucial that evidence is obtainable to specifically identify local problems. Such evidence ought to be supplied by data from death registers, which are important for the generation of life tables that in turn yield valuable knowledge to better target interventions that can mitigate the impact of such diseases.

### Death registration and the sustainable development goals

The United Nations Sustainable Development Goals (SDGs) have provided additional impetus for strengthening the CRVS by countries. The SDGs have 23 health-related targets and 67 health indicators, several of which are dependent on the CRVS for performance measurement [23,24]. There are also several other targets that require data from the CRVS for countries to calculate these indicators [25]. Table 1 lists the various SDG mortality indicators that rely on the CRVS for measurement.

**Table 1.** SDG mortality indicators dependent on the CRVS.

Indicator Number	SDG Indicator
3.1.1	Maternal Mortality Ratio
3.2.1	Under-Five Mortality Rate
3.2.2	Neonatal Mortality Rate
3.4.1	Mortality due to NCDs
3.4.2	Suicide Mortality Rate
3.6.1	Death from Road Traffic Injuries
3.9.1	Mortality due to air pollution
3.9.2	Mortality due to unsafe WASH services
3.9.3	Mortality due to unintentional poisoning
8.8.1	Occupational Injury Mortality
13.1.2	Mortality due to disasters
16.1.1	Homicides
16.1.2	Mortality due to conflicts
17.19.2	Death registration

Source: World Health Statistics: 2017

Given that the attainment of the SDGs requires multisectoral implementation approaches, it potentially confers some challenges to performance measurement as multiple stakeholders – with different and potentially conflicting measurement systems – are needed to effectively monitor the SDGs [26,27]. However, an efficient CRVS can transcend some of these constraints; its property of being capable to capture sum effects across multiple areas of interventions makes it particularly important to track SDGs progress.

Nigeria is the seventh largest country in the world with an estimated population of 191 million people [28]. At current fertility rates, the country is projected to become the third largest in the world by 2050 with a population of about 400 million after India and China [28]. With rapid population growth, the importance of efficient planning and the optimal allocation of limited resources to its most impactful areas cannot be overstated.

The disproportionately large contribution of Nigeria to the global burden of diseases is a compelling argument to prioritize the strengthening of its measurement systems, an important strategy and a global good towards the attainment of the SDGs; its continued underperformance will adversely affect the achievement of the global SDG targets [29,30]. Evaluating the progress towards achieving the SDGs will help in realigning efforts towards areas that require added attention. However, a recent assessment of the CRVS system in Nigeria concluded that the CRVS lacks the capacity to produce reliable mortality statistics for monitoring the SDGs [31].

## Methods and structure of the review

This paper utilized a systematic review of the literature, publicly available reports, and published laws of Nigeria that pertain CRVS, death registration in particular. We used specific search terms and applied explicit inclusion and exclusion criteria to determine which papers to retain for analyses. The paper also drew on authors’ firsthand knowledge and perspectives on the performance, gaps, and problems associated with the country’s death registration subsystem.

**Figure 1.**
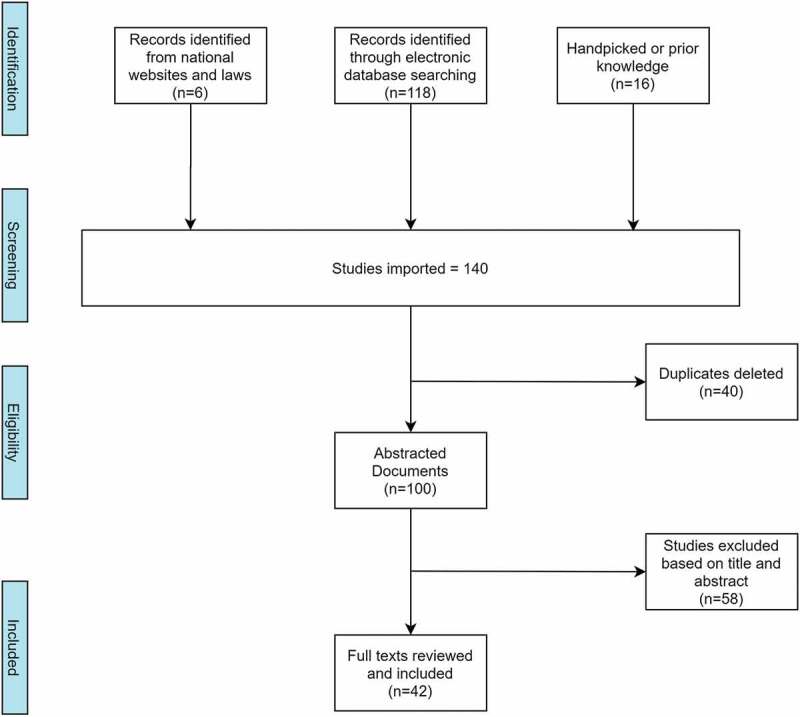
Systematic selection of studies on death registration in Nigeria.

In July 2020, utilizing the search terms (‘Death Registration’ AND Nigeria), (‘Death Certificates’ AND Nigeria) and (‘Coroners OR “Medical Examiners”’ AND Nigeria), we searched the websites of Nigerian government institutions including the National Population Commission, National Bureau of Statistics, and National Identity Management Commission for relevant reports and laws on death registration and on its completeness across the country. We also searched the databases of National Library of Medicine (PubMed), SCOPUS, Web of Science, and ProQuest (without a year limit) as well as the websites of the World Health Organization, World Bank, and United Nations Development Programme for relevant publications on death registration completeness in Nigeria. In addition, we relied on authors’ knowledge of unpublished sources. Journal articles that discussed the CRVS in Nigeria were screened in full. We included papers when they satisfied the criterion of discussing death registration in Nigeria, certification of the cause of death and/or challenges with the death certification and CRVS in the country. A total of 140 papers were initially included. We excluded papers when the challenges of the CRVS or death certification in Nigeria were neither identified nor when death registration performance in the country was not discussed. In all, 42 papers satisfied our inclusion and exclusion criteria and were retained for detailed analyses (see Figure 1).

We reviewed laws and policies on CRVS, extracted data on death registration completeness rate and other quality parameters from identified studies, extracted information on the challenges to death registration and potential actions that could help mitigate these challenges and critically assessed the problem from our collective knowledge of the CRVS system and the associated problems in Nigeria. We examined the institutional structure, leadership, and the law governing the process of death registration in Nigeria and the performance of death registration as well as challenges associated with death registration completeness in Nigeria.

Table 2 provides a list of the documents identified and reviewed.

**Table 2.** List of reviewed papers included by author and year of publication.

Government Websites and Laws of Nigeria	Published Literature (PubMed, ProQuest, Web of Science)	Hand Picked/Prior Knowledge/References of articles reviewed
[57]	[2]	[64]
[32]	[39]	[65]
[33]	[41]	[51]
[34]	[40]	[53]
[44]	[48]	[58]
[35]	[49]	[59]
	[50]	[60]
	[52]	[43]
	[42]	[37]
	[54]	[9]
	[55]	[63]
	[56]	[45]
	[36]	[46]
	[31]	[47]
	[66]	[68]
	[38]	[69]
	[61]	
	[62]	
	[67]	
	[18]	

## Results

Of the 42 papers that were reviewed, seven identified the different institutions responsible for managing the CRVS including death registration [32–38], 10 discussed the performance of death registration in Nigeria [9,39–47], 16 identified the challenges with cause of death certification [48–63], and 10 discussed issues with the CRVS in general [2,18,31,36,64–69].

### Institutional structure and leadership

The Nigeria Birth and Deaths etc. (Compulsory Registration) Act of 1992 was promulgated to provide a legal framework to institutionalize and compel the registration of vital events [32]. This Act superseded a decree written in 1989 primarily for the establishment of the National Population Commission [38]. The Act instituted the establishment of the National Population Commission and formally established the position of a Registrar-General for the country. The Registrar-General is expected to ‘exercise the powers and perform the duties conferred or imposed under or pursuant to this Act [32].’ The Act further pronounced the establishment of offices of the Chief Registrar in each state and the Federal Capital Territory (FCT) to perform roles at state-level equivalent to those carried out by the Registrar-General at the national level. State Chief Registrars report to the Registrar-General. It also established the offices of the Deputy-Chief Registrar for each Local Government Area within each state and the FCT. Registrars of various cadres were subsequently appointed to work under the supervision of the Deputy-Chief Registrars in Wards and registration points within the Local Government Areas. However, in 2007, the establishment of the National Identity Management Commission (as an independent federal institution separate from the National Population Commission) through an Act enabled this new commission with some responsibilities that were already handled by the National Population Commission [34].

The National Identity Management Commission was mandated to establish a national identity database with the commission responsible for maintaining the database, the registration of individuals, the issuance of multipurpose identity cards and for related matters. Among the responsibilities outlined in the National Identity Management Commission Act (Part II – Functions and Powers of the Commission), is the registration of births and deaths [34]. Besides the mention of a representative of the National Population Commission serving as one of the board members of the National Identity Management Commission, there are no other references made to the National Population Commission in the Act or how the two institutions would avoid duplication of effort.

Nigeria is a federation with three tiers of government: Federal, State, and Local Government [33]. The national constitution specifies the role of the National Population Commission as ‘to advise the President on population matters’ [33]. It also grants some level of autonomy to the different levels of government, including the responsibility of the local government authorities to register births, deaths, and marriages. As well as the two federal institutions described above, local government councils (774 of them across the country) also have independent processes for the conduct of vital registration within their local governments’ operations that amount to duplication of effort, confusion, and fragmentation of data and resources [36]. The consequence of multiple institutions with overlapping responsibilities is that the collation of statistics on death by each government agency is inaccurate with some deaths not at all reported while others may be reported more than once to different registration entities.

### Process of death registration

**Figure 2.**
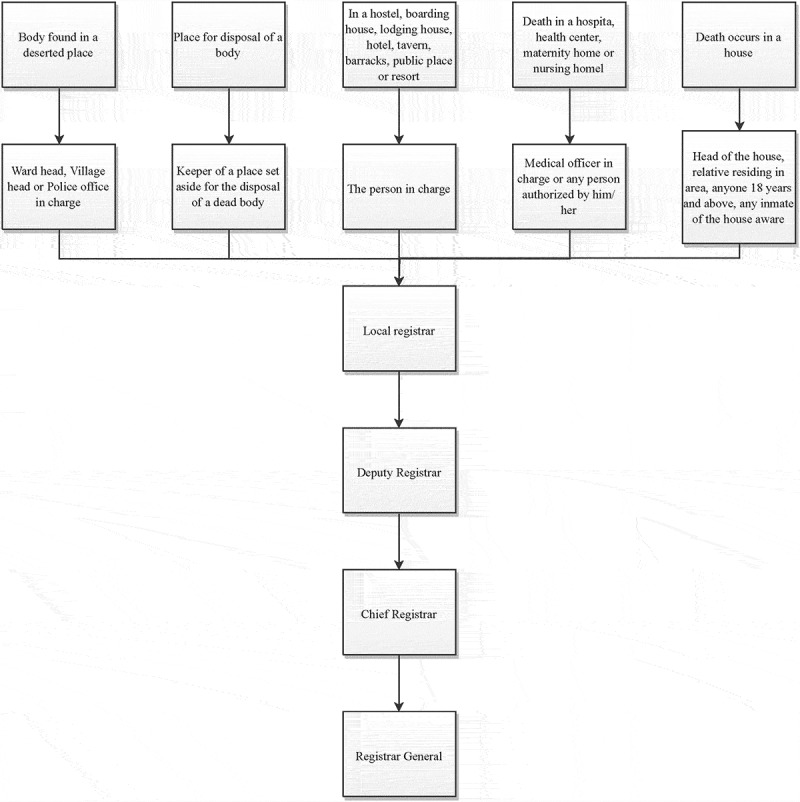
Process of death registration as described in births, deaths etc Compulsory Registration Act.

The process for death registration in Nigeria is provided for in the Birth and Deaths etc. (Compulsory Registration) Act of 1992 as modeled in Figure 2. The Act specifies persons responsible for reporting deaths and providing the required information at the point of registration. Deaths are to be reported to the local registrar of the locale where the incident occurred by the household head or a close family member if the death occurs at home, by a medical officer or any other authorized person if the death occurs in a health facility, and by the person in charge of a non-residential facility (hostel, barracks, hotel, etc.) if the death occurs there [32]. When a dead body is found in a public place, the ward or village head is required to report such a find. Deaths are required to be reported within 48 hours of its occurrence or discovery of the body. However, it has been observed that registration centers are occasionally not located close enough to those that need to report vital events; this burden of non-proximity of centers dissuades reporting or increase the likelihood of non-reporting [64]. Although many deaths occur in the health facilities, and there is a clearly defined responsible person for reporting the incident according to Nigerian laws [32], these facilities are poorly integrated with the National Population Commission for the purposes of death registration in Nigeria. Recent interventions have been carried out to relocate registration points within government-owned primary health facilities in rural communities across two states aimed at improving civil registration completeness [65].

Medical personnel that were the last to provide care to a deceased person whose death occurred outside a health facility are expected to certify a person dead with a properly completed medical certificate stating the cause of death [32]. In such instances when the doctor is not in a legal position to issue a death certificate, the coroner is expected to examine the body, determine the cause of death and issue a death certificate [32,35]. Until this is done, the burial certificate is not meant to be issued. The dictates of the law are however easily bypassed, as family members who qualify to act as informants in the death registration process can independently go to the National Population Commission office to register the fact of death, and obtain a burial certificate without a medical certification, or a coroner’s examination. Worse still, they can boycott the process entirely and not register the death at all.

### Performance of death registration

There was a dearth of death statistics on the website of the National Population Commission or across studies reviewed. There was no data reported for Nigeria in the World Health Organization database between 2008 and 2017, nor was there any submitted by the country according to a study that collated data between 1990 and 2005 [43]. The performance of CRVS in Nigeria between 1980 and 2012 has been poorly rated, which scored below 0.1 out of a maximum score of 1 on the VSPI [9]. The National Population Commission estimated that about 13.5% of deaths were registered in 2007 using survey data [44]. A country profile published on the website of IDRC’s Center for Excellence in CRVS in 2019 stated that death registration completeness was 10% in 2017 [47]. This statistic was noted to have been obtained through a questionnaire completed by the National Population Commission. There were no official government data on completeness reported at state or regional levels. The statistics identified were estimates produced by researchers, and lacked government involvement. For example, Ayeni & Olayinka in an evaluation of a special vital registration system conducted between 1964 and 1974 in Igbo-Ora (a suburb in Oyo State with a population of about 92,000 in 2017) reported high completeness rates of 87%, Akesode in 1980 reported that only 1.7% of deaths were registered in the old Western Region (the Western Region – before being delineated into states in 1976 – comprised of Lagos, Ogun, Ondo and Oyo states with a population of about 22 million according to the 2006 Population Census), while Adekolu-John reported no death registration in the Kainji lake area (a suburb in Niger state) in 1988. Tobin et al in 2013 reported about 39% death registration completeness in Egor Local Government Area of Edo state (with a population of about 340,000) and Williams in 2014 reported 21% in Mokola area of Ibadan, Oyo state [39,40,42,45,46].

### Challenges with death registration in Nigeria

The challenges with death registration in Nigeria affect both death certification and death registration, resulting in the poor VSPI scores [17]. These challenges are further elaborated below.

### Poor enforcement of national laws and policies

#### Births, deaths etc. (Compulsory Registration) Act

Notwithstanding the legal requirement that all deaths be compulsorily registered in Nigeria, deaths are still not being properly documented [2,46]. Factors associated with noncompliance with death registration include failure to enforce death registration as a precondition for burials, since ordinances permit burials in homes in addition to government-approved cemeteries, which make the enforcement of compliance with death registration difficult [53].

#### The coroner’s law

A coroner’s inquest is requisite for determination of cause and manner of death in suspicious deaths [32,35]. In such instances, it may become necessary to conduct an autopsy in accordance with the coroner’s law [57]. Sometimes including in hospitals, deciding on a definitive cause of death could be sufficiently unclear to warrant an autopsy [49,50,61]. In a study conducted by Lagos University Teaching Hospital, about 11% of 371 autopsies conducted on deaths labeled a priori as maternal deaths, the causes of death as determined by autopsy were unrelated to the initially stated causes of death [54]. In another study that reviewed 143 autopsies at the University of Benin Teaching Hospital, the concordance between clinical diagnosis and autopsy findings was only 54% [55]. Despite the importance of post-mortem examinations for definitive cause of death determination, the next of kin of patients often do not consent to autopsies based on their cultural and religious beliefs [52,53]. Consequently, autopsies are rarely conducted for strictly clinical or educational purposes since family consent is required. The majority of autopsies conducted in Nigeria are for medico-legal reasons. A study in north-central Nigeria reported that 96% of the autopsies conducted were for this purpose [60]. Another study from southern Nigeria reported that 89% of the 5035 autopsies performed over a 20-year period in the facility were coroner’s cases [48]. In the FCT, 65 (70%) of autopsies conducted within a two-year period were coroner’s cases [56]. The higher proportion of autopsies related to coroner’s cases aside from being a legal mandate is likely associated with unresolved homicide cases under active police investigation. Despite this possibility, they are not always performed. Conflicts between health workers and the family of the deceased have been known to arise in situations when hospital personnel have insisted on the need for a coroner’s autopsy prior to issuance of a death certificate. Disruption of services and assault of staff by the families of the deceased arising from such conflicts are not uncommon.

Based on the experience of the authors, another factor affecting coroners’ autopsies is the fees charged to the family of deceased which is contrary to law. Coroners’ autopsies are to be funded by the government through the Ministry of Justice. In all states of the federation, coroners’ autopsies require surviving family members to pay up. In many instances, the grieving family may be expected to pay as much as two hundred thousand naira (about US$600) or higher. When families decline an autopsy, as is inevitable for many, another barrier to death certification is created with health workers surrendering unexamined corpses with undetermined cause of death to relatives for burial.

### Inadequate human and financial resources

The cost of maintaining an efficient CRVS is high and remains an influencing factor for the poor performance of the CRVS in Nigeria [2,65]. However, non-investment in an adequate CRVS also has negative consequences as appropriate evidence to inform planning is not readily available. An efficient health information system requires long-term investment including for social and economic data that need to be harmonized with other data from the health sector [37]. The multiple parallel institutions established for CRVS fragments the overall amount of resources available to efficiently fund the CRVS in the country [36]. This results in high overhead costs across multiple institutions that working together in a single institution could eliminate.

There is evidence that doctors in Nigeria are poorly trained on the completion of death certificates in accordance with current guidelines, contributing to poor quality of cause of death reporting. Seventy-one percent (71%) of respondent physicians in a survey reported that they had never received training on the completion of death certificate forms [59]. Similarly, 96% of final year medical students at the Lagos University Teaching Hospital reported that they had not received formal training on how to complete death certificates [58]. In another study, fewer than 50% of doctors could identify seven reasons for the referral for a coroner’s examination [59]. The above, coupled with Nigeria’s high population to doctor ratio means the needed manpower and know-how to provide certification of death in accordance with global standards is not readily available in the country. When death certificates are not properly completed, or the cause of death is unknown or not determined, whilst a death might be registered, it might not be accompanied by accurate/any information on cause of death. These affect quality of cause of death reporting, as well as level of cause-specific detail.

### Political issues

Poor demand and use of products from the CRVS system by policymakers negatively affect its overall performance as they are less likely to pay attention to, or allocate adequate resources to the system [36,37,69].

### Religious, cultural and socio-economic issues

Religion and culture are major factors affecting death certification in Nigeria [53,60]. According to the Islamic doctrine practiced predominantly in the northern part of the country, the body must be interred on the day of death if the death occurred before sunset or within the next 24 hours if death occurred after sunset [51,53]. The time needed for the burial rites to be conducted is often too short for an appropriate cause of death investigation to be carried out if it is not readily known. Whereas the practice of burying the deceased in Muslim communities is well established with its tendency to reduce demand for autopsies, the practice in some non-Muslim communities in the country however may not differ much, albeit for different reasons. Here the tendency to bury the dead soon or immediately after death appears rooted in culture, which may be connected to socio-economic conditions. In most rural settings, doctors and hospitals are scarcely available. Many sick persons are not hospitalized and most die at home unattended by medical doctors. As a result, most deaths are neither reported nor certified; and the dead are buried without delay. In this setting, whereas people may be less rigid and more likely to cooperate if an autopsy is advised, family members and communities are likely to be reluctant to deviate from what has been accepted as the ‘norm’. Deaths generally come with negative emotions especially when the deceased is very young in age and is considered not to have lived a full or accomplished life. As such, deaths at young ages may not be registered because the act of registration is viewed as a reminder of the deceased’s short unfulfilled life. There are also many superstitious beliefs about the death of young people, especially during infancy in different parts of the country [42,45,70]. The Yorubas and Igbos from the southern part of Nigeria believe in the existence of some children referred to as ‘Abiku’ by the Yorubas or ‘Ogbanje’ by the Igbos. An *Abiku or Ogbanje* child is born and destined to die unless some rituals are performed [63]. Based on the belief that the child belongs to a group of spirit children whose purpose is unfulfilled, these societies prescribe that the short existence of the child should neither be discussed nor recognized when deceased, proscribing death registration and certification of cause of death [36,70]. As a result, once a child is branded as being an *Abiku*, they are treated differently and discriminated against by the family and community. Should the child eventually die, their deaths are less likely to be reported to the authorities.

### Technology-related factors

Poor adoption of technology by the National Population Commission remains a major challenge. An earlier report stated that: ‘ICT is used sparingly in its operation especially at the basic operation level the registration centers’, thus creating a major barrier to efficiency [36]. This finding adversely affects the ability of the National Population Commission to deliver on its mandate including on death registration across the country.

## Discussion

There was paucity of data on the level of completion of death registration in Nigeria and the last published national report on death registration in the country was in 2007. The Demographic and Health Surveys and Multiple Indicator Cluster Surveys have served as the major alternate sources of vital statistics data, but these data sources lack information on death registration. There was only one documented effort to conduct a follow-up cause of death determination by verbal autopsy which followed the 2013 DHS [71,72]. However, there was evidence of the use of this method in the country on smaller scale studies as far back as 1996 [73].

CRVS needs to be seen and implemented as part of public health, a means to sustainable development, investment, and good governance as well as a human rights issue [74]. The African Union has spearheaded a drive by African countries to improve CRVS for monitoring development goals [25]. A commendable example is the Africa Program on Accelerated Improvement of CRVS (APAI-CRVS) with the goal of harmonizing different CRVS programs into a unified framework (http://www.apai-crvs.org/). The APAI-CRVS has developed guidelines to improve death registration in Africa [75]. There is also the African Symposium on Statistical Development (ASSD) which organized five symposia on CRVS between 2011 and 2015. Consequently, more than half of 44 countries that participated have seen appreciable progress in CRVS systems [75]. In 2015, the African Union adopted Agenda 2063, a development framework to achieve accelerated, sustained, and inclusive economic growth [25]. There has been a recognition that sustainable development could not be properly verified without a functional CRVS [24]. In furtherance of this effort, in July, 2016, the Union also declared 2017–2026 as ‘A Decade for repositioning Civil Registration and Vital statistics (CRVS) in Africa’s continental, regional and national development agenda’ [75]. Nigeria and other members of the African Union are obligated to respond to the declaration with appropriate actions.

In line, with this review and the shortcomings identified, we have outlined important actions that can help reposition the CRVS in the country: There is an urgent need to review laws and parallel institutions that have been established for CRVS management. These institutions can be streamlined to improve efficiency and their resources combined to increase the resources available for CRVS. The review of laws should clearly delineate roles and avoid duplication of responsibilities at all levels. From the analysis by Maduekwe et al. (2017), it is evident that a distributed responsibility of CRVS management is the most efficient way that the system can attain its objectives in Nigeria [36]. The National Population Commission should work with the States and Local Government Authorities who should take charge of the registration responsibilities within their geographic area rather than the National Population Commission establishing registration points across all the 774 local governments in the country. The recent guidelines published by the UN Statistics Division titled ‘Handbook on civil registration, vital statistics, and identity management systems: Communication for Development’ stresses the importance of having a one-identity approach in a country [76]. An effort to improve coordination between the Federal Ministry of Health and other health data generating institutions, including the National Population Commission, with the establishment of a National Health Data Governance Council can also be leveraged to improve coordination [77].

Advocating and educating policymakers (including federal, state, and LGA legislators) on the benefits of the CRVS and its potential for taking the place of periodic censuses. With improved knowledge on its important and potential use, such can create a push for local politicians to raise awareness on birth and death registration compliance in their communities.

There is a need for the adoption and leveraging of advances in Information and Communications Technology that will facilitate the distributed system as previously suggested. This system will enable Local Government Registrars to submit records to the National civil registration database managed by the National Population Commission without the National Population Commission establishing parallel structures (as is currently done) in every local government that duplicates the responsibilities of the local government employed registrars.

Established laws and policies such as ensuring burials, cremations, or other means of body disposal do not take place without death registration and burial approvals should be enforced. This can be achieved by ensuring that burials take place in only government-approved cemeteries and body disposal facilities. However, this needs to be preceded by a pronouncement of a ban of burials outside of government-approved cemeteries and extensive public education.

There is a need for training on medical certification of the cause of death for medical workers who are primarily responsible for certifying deaths. Medical curricula also need to be adjusted to ensure that medical students are trained and confident about executing the necessary steps leading to the certification of cause of death and completion of death certificates. In addition, continuous linkages that help ensure all reported facts of death are properly certified by a medical practitioner should be encouraged.

Establishment of a financing mechanism that funds post-mortem examination for deceased people when necessary without the expectation for families to cover the cost is needed. Since Nigeria is currently making policy effort towards achieving Universal Health Coverage [78], the importance of knowing the cause of death of people will be of increasing importance. As such, the national strategy can embed some resources towards ensuring that the cost of post-mortem examinations is covered where necessary. In addition, State Ministries of Justice can ensure the availability of a medical examiner for each local government area to facilitate the ease of certification of death. Furthermore, the utilization of recent advances in cause of death determination such as verbal autopsy and MITS can be embedded into the national standard for cause of death determination [12,79,80]. While verbal autopsy can help fill the gap in information on cause of death, it requires significant time for the completion of the necessary interviews. MITS has also emerged as an important opportunity to fill the gap on cause of death with a higher level of precision than verbal autopsy using tissue diagnosis [12]. Use of MITS and verbal autopsy for characterizing cause of death remains underdeveloped in Nigeria.

Theory-informed and data-based social and behavior change interventions targeting the general population and that rely on strategic communication and other approaches to promote compliance with CRVS are needed. This can include illustrative messages using real-life scenarios as suggested for birth registration using football stars [81]. Messages for the general population should address the potentially modifiable individual, household, and community factors that affect registration of vital events. Furthermore, advocacy towards religious and community leaders should be part of a comprehensive social and behavior change approach.

Sample Registration Systems can be considered as interim measures for addressing the poor CRVS. The Igbo-Ora surveillance site which produced very high completeness of death rates in the 1960s and 1970s, the recently introduced Nahuche Demographic Surveillance Site and the Cross River Health and Demographic Surveillance Systems are important examples of Sample Registration Sites that can be leveraged upon to provide accurate statistics from where national projections can be done [42,82,83]. However, the state of the Igbo-Ora project is not well known and the more recent Nahuche demographic surveillance site is threatened by inadequate funding [84]. There is limited information in the literature on the level of viability of the Calabar site as well. All the same, these efforts need to be adequately planned for and funded by the government.

### Study limitations

Death registration completeness in Nigeria has seldom been studied, and the few studies identified covered limited geographic sections of the country. As a result, there was limited evidence to determine the national level of performance of death registration. We utilized our collective knowledge and field experiences to identify several challenges with death registration in Nigeria. This could have introduced some bias in the discussions and conclusions reached.

## Conclusion

Death registration is an important component of the CRVS and of particular importance in the measurement of progress towards the SDGs and for routine planning. It is, however, challenged by poor enforcement of laws, duplicated responsibilities in management of civil registration data, inadequate resources and poor adoption of technology. Addressing the challenges with death registration in Nigeria requires a holistic approach which will include revision of laws that will ensure streamlining of institutions, leverage available resources and make room for enforcing laws that will drive registration of these vital events. In order to streamline institutions, the Federal Government will have to enact legislation that will make the local government responsible for the management of registration processes which is coordinated and standardized by the National Population Commission/National Identity Management Commission. All other laws that establish parallel institutions to collect CRVS data must be repealed in the process. Enhancing the CRVS system should be fostered by electronic systems that support hierarchical relationship with all stakeholders contributing data into a single electronic archive that will eliminate duplication enabled by the duplicity of institutions running parallel processes.

## Paper context

Only 10% of deaths were registered in Nigeria’s Civil Registration and Vital Statistics (CRVS) system in 2017. Multiple government agencies share responsibility for death registration with poor coordination across them. Civil registration laws on death registration are poorly enforced compounded by burials permitted in homes. There is need to streamline the laws establishing agencies on CRVS, improve coordination and funding and embed technology in CRVS processes.

## References

[1] United Nations. Principles and recommendations for a vital statistics system. Revision 2 [Internet]. New York: United Nations Publications; 2001 [cited 2014 428]. Available from: http://unstats.un.org/unsd/publication/SeriesM/SeriesM_19rev2E.pdf

[2] Adedini SA, Odimegwu CO. Assessing knowledge, attitude and practice of vital registration system in South-West Nigeria. Ife Psychol. 2011;19:456–13.

[3] World Health Organization, World Bank. Global civil registration and vital statistics: scaling up investment plan 2015–2024 [Internet]. The World Bank; 2014 [cited 2015 727]. p. 1–92. Report No.: 88351. Available from: http://documents.worldbank.org/curated/en/2014/05/19581045/global-civil-registration-vital-statistics-scaling-up-investment-plan–2015–2024

[4] Griffith R. Legal issues of death. Care of the dying and deceased patient [Internet]. Wiley-Blackwell; 2009 [cited 2018 817]. p. 193–222. Available from: https://0-onlinelibrary-wiley-com.innopac.wits.ac.za/doi/abs/10.1002/9781444315271.ch10

[5] United Nations Economic Commission for Africa. Death registration [Internet]. 2019 [cited 2020 717]. Available from: https://www.uneca.org/sites/default/files/uploaded-documents/Statistics/CRMC3/death_registration_en.pdf

[6] Setel PW, Macfarlane SB, Szreter S, et al. A scandal of invisibility: making everyone count by counting everyone. Lancet. 2007;370:1569–1577. 17992727 10.1016/S0140-6736(07)61307-5

[7] Garenne M, Collinson MA, Kabudula CW, et al. Improving completeness of birth and death registration in rural Africa. Lancet Glob Health. 2016;4:e604–e605. 27539803 10.1016/S2214-109X(16)30146-2

[8] Mbondji PE, Kebede D, Soumbey-Alley EW, et al. Health information systems in Africa: descriptive analysis of data sources, information products and health statistics. J R Soc Med. 2014;107:34–45. 10.1177/0141076814531750 PMC4109358 24914127

[9] Mikkelsen L, Phillips DE, AbouZahr C, et al. A global assessment of civil registration and vital statistics systems: monitoring data quality and progress. Lancet [Internet]. 2015 [cited 2015 516];386:1395–1406. Available from: http://linkinghub.elsevier.com/retrieve/pii/S0140673615601714 10.1016/S0140-6736(15)60171-4 25971218

[10] Salzberg NT, Sivalogan K, Bassat Q, et al. Mortality surveillance methods to identify and characterize deaths in child health and mortality prevention surveillance network sites. Clin Infect Dis. 2019;69:S262–S273. 31598664 10.1093/cid/ciz599 PMC6785672

[11] Cunningham SA, Shaikh NI, Nhacolo A, et al. Health and demographic surveillance systems within the child health and mortality prevention surveillance network. Clin Infect Dis. 2019;69:S274–S279. 31598663 10.1093/cid/ciz609 PMC6785673

[12] Taylor AW, Blau DM, Bassat Q, et al. Initial findings from a novel population-based child mortality surveillance approach: a descriptive study. Lancet Glob Health. 2020;8:e909–e919. 32562647 10.1016/S2214-109X(20)30205-9 PMC7303945

[13] Adair T, Lopez AD. Estimating the completeness of death registration: an empirical method. PLoS One. 2018;13:e0197047. 29847573 10.1371/journal.pone.0197047 PMC5976169

[14] World Health Organization. Civil registration: why counting births and deaths is important [Internet]. World Health Organization; 2014 [cited 2019 226]. Available from: https://www.who.int/news-room/fact-sheets/detail/civil-registration-why-counting-births-and-deaths-is-important

[15] Lopez AD, Setel PW. Better health intelligence: a new era for civil registration and vital statistics? BMC Med. 2015;13:73. 25885782 10.1186/s12916-015-0333-4 PMC4387703

[16] Murray CJL, Rajaratnam JK, Marcus J, et al. What can we conclude from death registration? Improved methods for evaluating completeness. PLoS Med [Internet]. 2010 [cited 2018 1010];7:e1000262. Available from: https://www.ncbi.nlm.nih.gov/pmc/articles/PMC2854130/10.1371/journal.pmed.1000262 PMC2854130 20405002

[17] Phillips DE, Lozano R, Naghavi M, et al. A composite metric for assessing data on mortality and causes of death: the vital statistics performance index. Popul Health Metr. 2014;12:14. 24982595 10.1186/1478-7954-12-14 PMC4060759

[18] Feyi-Waboso P. SDGs: the need for vital registration and accurate record keeping. Afr J Reprod Health [Internet]. 2017 [cited 2018 821];20. Available from: https://www.ajrh.info/index.php/ajrh/article/view/164 10.29063/ajrh2016/v20i3.8 29553193

[19] Mahapatra P, Shibuya K, Lopez AD, et al. Civil registration systems and vital statistics: successes and missed opportunities. Lancet. 2007;370:1653–1663. 18029006 10.1016/S0140-6736(07)61308-7

[20] Phillips DE, AbouZahr C, Lopez AD, et al. Are well functioning civil registration and vital statistics systems associated with better health outcomes? Lancet. 2015;386:1386–1394. 25971222 10.1016/S0140-6736(15)60172-6

[21] Redfern P. Which countries will follow the Scandinavian lead in taking a register-based census of population? J Off Stat. 1986;2:415. 12341360

[22] Omran AR. The epidemiologic transition: a theory of the epidemiology of population change. Milbank Q. 2005;83:731–757. 16279965 10.1111/j.1468-0009.2005.00398.x PMC2690264

[23] Thomas JC, Silvestre E, Salentine S, et al. What systems are essential to achieving the sustainable development goals and what will it take to marshal them? Health Policy Plan. 2016;31:1445–1447. 27296063 10.1093/heapol/czw070

[24] World Health Organization. World Health Statistics 2017: monitoring health for the SDGs, Sustainable Development Goals [Internet]. Geneva, Switzerland; 2017. Available from: http://apps.who.int/iris/bitstream/10665/255336/1/9789241565486-eng.pdf?ua=1

[25] African Union. Civil registration and vital statistics for monitoring the progress made in implementing agenda 2063 and the 2030 agenda for sustainable development. African Union Commission; 2017. Report No.: AUC/CRMC4/2017/2.

[26] Mills SA, Carla Kim J, Rassekh BM, et al. Civil registration and vital statistics for monitoring the sustainable development goals [Internet]. World Bank; 2017 [cited 2018 42]. Available from: https://elibrary.worldbank.org/doi/abs/10.1596/27533

[27] Nabyonga-Orem J. Monitoring Sustainable Development Goal 3: how ready are the health information systems in low-income and middle-income countries? BMJ Glob Health. 2017;2:e000433. 10.1136/bmjgh-2017-000433 PMC5663251 29104767

[28] Population Reference Bureau. 2017 world population datasheet [Internet]. 2017 [cited 2018 15]. Available from: http://www.prb.org/pdf17/2017_World_Population.pdf

[29] Lozano R, Naghavi M, Foreman K, et al. Global and regional mortality from 235 causes of death for 20 age groups in 1990 and 2010: a systematic analysis for the Global Burden of Disease Study 2010. Lancet. 2012;380:2095–2128. 23245604 10.1016/S0140-6736(12)61728-0 PMC10790329

[30] Wang H, Naghavi M, Allen C, et al. Global, regional, and national life expectancy, all-cause mortality, and cause-specific mortality for 249 causes of death, 1980–2015: a systematic analysis for the Global Burden of Disease Study 2015. Lancet. 2016;388:1459–1544. 27733281 10.1016/S0140-6736(16)31012-1 PMC5388903

[31] Maduekwe NI, Banjo OO, Sangodapo MO. Data for the sustainable development goals: metrics for evaluating civil registration and vital statistics systems data relevance and production capacity, illustrations with Nigeria. Soc Indic Res. 2018;140:101–124.

[32] Federal Government of Nigeria. Births, deaths etc (Compulsory Registration) Act. 1992.

[33] Federal Government of Nigeria. Constitution of the Federal Republic of Nigeria 1999: [Internet]. 1999 [cited 2017 64]. Available from: http://www.nigeria-law.org/ConstitutionOfTheFederalRepublicOfNigeria.htm

[34] Federal Government of Nigeria. The National Identity Management Commission Act, 2007 [Internet]. 2007 [cited 2016 125]. Available from: http://www.nimc.gov.ng/sites/default/files/reports/nimc_act.pdf

[35] Lagos State Government. Coroner’s system law: a law to establish the Lagos state coroner’s system, regulate the process of death investigation and for connected purposes. Lagos, Nigeria: Lagos State Government; 2007.

[36] Maduekwe NI, Banjo OO, Sangodapo MO. The Nigerian civil registration and vital statistics system: contexts, institutions, operation. Soc Indic Res. 2017;134:651–674.

[37] Meribole EC, Makinde OA, Oyemakinde A, et al. The Nigerian health information system policy review of 2014 : the need, content, expectations and progress. Health Info Libr J. 2018;35:285–297. 30417971 10.1111/hir.12240

[38] Nigeria. National Population Commission Decree 1989 (No. 23 of 1989), 19 October 1989. Annu Rev Popul Law. 1989;16:4. 12344406

[39] Adekolu-John EO. A study of vital and health statistics of the Kainji Lake Area of Nigeria. Afr J Med Med Sci. 1988;17:149–156. 2845754

[40] Akesode FA. Registration of births and deaths in Lagos, Nigeria. J Trop Pediatrics. 1980;26:150–155. 10.1093/tropej/26.4.150 7463552

[41] al-Haddad BJS, Jedy-Agba E, Oga E, et al. Comparability, diagnostic validity and completeness of Nigerian cancer registries. Cancer Epidemiol. 2015;39:456–464. 25863982 10.1016/j.canep.2015.03.010 PMC4446152

[42] Ayeni O, Olayinka A. An evaluation of a special-type vital statistics registration system in a rural area of Nigeria. Int J Epidemiol. 1979;8:61–68. 489226 10.1093/ije/8.1.61

[43] Mathers CD, Ma Fat D, Inoue M, et al. Counting the dead and what they died from: an assessment of the global status of cause of death data. Bull World Health Organ. 2005;83:171–177c. 15798840 PMC2624200

[44] National Population Commission, Federal Republic of Nigeria. Report on livebirths, deaths & stillbirths registration in Nigeria (1994–2007) [Internet]. Abuja, Nigeria: National Population Commission; 2008 [cited 2014 429]. Available from: http://population.gov.ng/images/Report%20on%20Birth-Death-Stillbirth-Registration.pdf

[45] Tobin EA, Obi AI, Isah EC. Status of birth and death registration and associated factors in the South-south region of Nigeria. Ann Nigerian Med [Internet]. 2013 [cited 2014 418];7. Available from: http://www.anmjournal.com/text.asp?2013/7/1/3/119979

[46] Williams AO. Assessment of the completeness of births and deaths registration in an urban Nigerian community. Etude Popul Afr [Internet]. 2014;27. Available from: https://search.proquest.com/docview/2166116505?accountid=15083

[47] Centre of Excellence for CRVS Systems. Snapshot of civil registration and vital statistics systems of Nigeria [Internet]. 2019 [cited 2020 716]. Available from: https://crvssystems.ca/country-profile/nigeria#footnote4_en3l66p

[48] Akhiwu WO, Nwafor CC, Igbe AP. A 20 year retrospective analysis of medicolegal deaths in a tertiary hospital setting in Nigeria. Niger J Clin Pract. 2013;16:535–539. 23974754 10.4103/1119-3077.116910

[49] Aligbe J, Akhiwu W, Nwosu S. Prospective study of coroner’s autopsies in Benin City, Nigeria. Med Sci Law. 2002;42:318–324. 12487517 10.1177/002580240204200407

[50] Amakiri C, Akang E, Aghadiuno P, et al. A prospective study of coroner’s autopsies in University College Hospital, Ibadan, Nigeria. Med Sci Law. 1997;37:69–75. 9029924 10.1177/002580249703700115

[51] Areo O. How cultural, religious beliefs hamper medical research in Nigeria, by NIMR [Internet]. 2017 [cited 2018 821]. Available from: https://guardian.ng/features/how-cultural-religious-beliefs-hamper-medical-research-in-nigeria-by-nimr/

[52] Arodiwe EB, Nwokediuko SC, Ike SO. Medical causes of death in a teaching hospital in South-Eastern Nigeria: a 16 year review. Niger J Clin Pract. 2014;17:711. 25385907 10.4103/1119-3077.144383

[53] Atanda AT, Umar AB, Yusuf I, et al. Autopsy and religion: A review of the literature. Sahel Med J. 2016;19:119.

[54] Daramola AO, Elesha SO, Banjo AAF. Medical audit of maternal deaths in the Lagos University Teaching Hospital, Nigeria. East Afr Med J. 2005;82:285–289. 16175778 10.4314/eamj.v82i6.9298

[55] Diegbe IT, Idaewor PE, Igbokwe UO. Autopsy audit in a teaching hospital in Nigeria–the Benin experience. West Afr J Med. 1998;17:213–216. 9814096

[56] Duduyemi BM, Ojo BA. Coroner’s autopsies in Nigeria Capital City of Abuja: a review of 65 consecutive cases. Indian J Med Forensic Med Toxicol. 2014;8:53–57.

[57] Federal Government of Nigeria. Coroners Act [Internet]. 1945. Available from: http://lawnigeria.com/LawsoftheFederation/BIRTHS,-DEATHS,-ETC.-%28COMPULSORY-REGISTRATION%29-ACT.html

[58] Inem VA, Izegbu MC. Accurate completion of death certificates: the need for formalised training in the Nigerian medical curriculum. Niger J Health Biomed Sci. 2005;4:76–81.

[59] Izegbu MC, Agboola AOJ, Shittu LAJ, et al. Medical certification of death and indications for medico-legal autopsies: the need for inclusion in continue medical education in Nigeria. Sci Res Essays. 2006;1:61–64.

[60] Mandong BM, Manasseh AN, Ugwu BT. Medicolegal autopsies in North Central Nigeria. East Afr Med J. 2006;83:626–630. 17455452 10.4314/eamj.v83i11.9480

[61] Nwafor CC, Igbe AP, Akhiwu WO. Study of natural causes of death in medicolegal autopsies seen in University of Benin Teaching Hospital. Niger Postgrad Med J. 2014;21:305–310. 26151985

[62] Obiorah CC, Amakiri CN. Review of population based coroners autopsy findings in Rivers state of Nigeria. Forensic Sci Int. 2013;233:1–6. 24314494 10.1016/j.forsciint.2013.08.008

[63] Ogunjuyigbe PO. Under-five mortality in Nigeria: perception and attitudes of the Yorubas towards the existence of “Abiku”. Demographic Res. 2004;11:43–56.

[64] Abbas AM. Effects of distance and population on birth registration coverage: an analysis of gombe state situation, Nigeria. Int J Innov Res Stud [Internet]. 2014 [cited 2014 427];3. Available from: http://www.ijirs.com/vol3_issue-2/39.pdf

[65] Adi AE, Abdu T, Khan A, et al. Understanding whose births get registered: a cross sectional study in Bauchi and Cross River states, Nigeria. BMC Res Notes. 2015;8:79. 25879591 10.1186/s13104-015-1026-y PMC4369829

[66] Makinde OA, Olapeju B, Ogbuoji O, et al. Trends in the completeness of birth registration in Nigeria: 2002–2010. Demographic Res. 2016;35:315–338.

[67] Adenomon MO, Anikweze EC. On the trends of registered birth and death rates in Nigeria: evidence from generalized linear models [Internet]. MDPI AG; 2019 [cited 2020 722]. Available from: https://search.proquest.com/docview/2268455059/abstract/6C7F49F6759748C2PQ/1

[68] Atama C, Igwe I, Odii A, et al. Contextual and normative challenges to vital registration in Nigeria. Int J Res Arts Social Sci. 2020;12:127–137.

[69] Ukoji UV, Okoronkwo E, Imo CK, et al. Civil registration and vital statistics as sources of socio-demographic data for good governance in Nigeria. Niger J Sociol Anthropol. 2019;17:102.

[70] Adebileje A. Socio-cultural and attitudinal study of selected Yoruba taboos in South West Nigeria. Stud Lit Lang. 2012;4:94–100.

[71] Adewemimo A, Kalter HD, Perin J, et al. Direct estimates of cause-specific mortality fractions and rates of under-five deaths in the northern and southern regions of Nigeria by verbal autopsy interview. PLoS One; San Francisco. 2017;12:e0178129. 28562611 10.1371/journal.pone.0178129 PMC5451023

[72] Koffi AK, Perin J, Kalter HD, et al. How fast did newborns die in Nigeria from 2009–2013: a time-to-death analysis using verbal/social autopsy data. J Glob Health [Internet]. [cited 2020 728];9. Available from: https://www.ncbi.nlm.nih.gov/pmc/articles/PMC6657661/10.7189/jogh.09.020501 PMC6657661 31360450

[73] Asuzu MC, Johnson OO, Owoaje EE, et al. Questions on adult mortality. World Health Forum. 1996;17:373–376. 9060234

[74] Brolan CE, Gouda HN, AbouZahr C, et al. Beyond health: five global policy metaphors for civil registration and vital statistics. Lancet. 2017;389:1084–1085. 28322806 10.1016/S0140-6736(17)30753-5

[75] APAI-CRVS. Making everyone visible in Africa [Internet]. 2017 [cited 2020 722]. Available from: http://apai-crvs.org/sites/default/files/public/Making%20Everyone%20Visible_September%20EN%20_0.pdf

[76] United Nations Statistics Division. Handbook on civil registration, vital statistics and identity management systems: communication for development [Internet]. 2019. Available from: https://unstats.un.org/unsd/demographic-social/Standards-and-Methods/files/Handbooks/crvs/CRVS-IdM-E.pdf

[77] Makinde OA, Oyediran KA. Is the Nigerian government one step closer to evidence-based decision making in health? [Internet]. 2017 [cited 2017 92]. Available from: http://nigeriahealthwatch.com/is-the-nigerian-government-one-step-closer-to-evidence-based-decision-making-in-health/

[78] Makinde OA, Sule A, Ayankogbe O, et al. Distribution of health facilities in Nigeria: implications and options for Universal Health Coverage. Int J Health Plann Manag. 2018;33:e1179–e1192. 10.1002/hpm.2603 30091473

[79] World Health Organization. WHO | verbal autopsy standards: ascertaining and attributing causes of death [Internet]. WHO. World Health Organization; 2019 [cited 2020 716]. Available from: http://www.who.int/healthinfo/statistics/verbalautopsystandards/en/

[80] Nichols EK, Byass P, Chandramohan D, et al. The WHO 2016 verbal autopsy instrument: an international standard suitable for automated analysis by InterVA, InSilicoVA, and Tariff 2.0. PLoS Med. 2018;15:e1002486. 29320495 10.1371/journal.pmed.1002486 PMC5761828

[81] Makinde OA, Odimegwu CO, OlaOlorun FM. A unique opportunity to use football to improve birth registration awareness and completeness in Nigeria. Br J Sports Med. 2018;52:1529–1530. 28455365 10.1136/bjsports-2016-097404 PMC6241617

[82] Alabi O, Doctor HV, Jumare A, et al. Health & demographic surveillance system profile: the nahuche health and demographic surveillance system, Northern Nigeria (Nahuche HDSS). Int J Epidemiol. 2014;43:1770–1780. 25399021 10.1093/ije/dyu197

[83] Arikpo I, Eteng I, Okoro A, et al. Improving the recording and reporting of facility-based mortality using open source mobile technology: lessons from Cross River HDSS, Nigeria. J Comput Inform Syst. 2016;20:27–36.

[84] Alabi O, Doctor HV, Afenyadu GY, et al. Lessons learned from setting up the Nahuche Health and Demographic Surveillance System in the resource-constrained context of northern Nigeria. Glob Health Action [Internet]. 2014 [cited 2015 423];7:23368. Available from: http://www.ncbi.nlm.nih.gov/pmc/articles/PMC4014660/10.3402/gha.v7.23368 PMC4014660 24809831

